# *catena*-Poly[diimidazolium [bis­(μ-pyridine-2,5-dicarboxyl­ato)bis­[diaqua­praseodymate(III)]]-bis­(μ-pyridine-2,5-dicarboxyl­ato)]

**DOI:** 10.1107/S1600536811012360

**Published:** 2011-04-13

**Authors:** Wenjun Zhang, Yanmei Chen, Tao Lei, Yahong Li, Wu Li

**Affiliations:** a Qinghai Institute of Salt Lakes, Chinese Academy of Sciences, Xining 810008, People’s Republic of China; b Key Laboratory of Organic Synthesis of Jiangsu Province, College of Chemistry and Chemical Engineering, Soochow University, Suzhou 215123, People’s Republic of China

## Abstract

The title compound {(C_3_H_5_N_2_)_2_[Pr_2_(C_7_H_3_NO_4_)_4_(H_2_O)_4_]}_*n*_, has a chain structure featuring a dimeric unit consisting of two Pr^III^ atoms within a dodecahedral environment. Each of the metal cations is coordinated by two N atoms and two O atoms from two pyridine-2,5-dicarboxyl­ate ligands, two O atoms from another two pyridine-2,5-dicarboxyl­ate ligands and two water O atoms. The Pr^III^ ions are bridged by two ligands along the *c* axis, forming the dimeric unit, and these are connected by four ligands along the *b* axis, forming a chain. N—H⋯O and O—H⋯O hydrogen bonds are found in the structure.

## Related literature

For praseodymium complexes with pyridine-dicarboxylate ligands, see: Chen *et al.* (2011 ▶); Zhao *et al.* (2009 ▶); Song *et al.* (2006 ▶); Chi *et al.* (2009 ▶). For complexes with similar structures, see: Li, Zhang *et al.* (2009 ▶); Li, Chen *et al.* (2009 ▶); Huang *et al.* (2009 ▶); Zhang *et al.* (2005 ▶, 2007 ▶). 
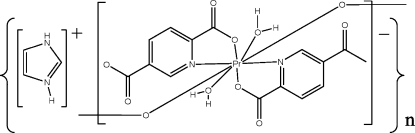


## Experimental

### 

#### Crystal data

(C_3_H_5_N_2_)_2_[Pr_2_(C_7_H_3_NO_4_)_4_(H_2_O)_4_]
*M* *_r_* = 1152.48
Triclinic, 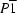

*a* = 9.5444 (19) Å
*b* = 10.667 (2) Å
*c* = 11.222 (2) Å
α = 64.63 (3)°
β = 79.50 (3)°
γ = 87.50 (3)°
*V* = 1014.3 (3) Å^3^
*Z* = 1
Mo *K*α radiation
μ = 2.47 mm^−1^
*T* = 293 K
0.19 × 0.16 × 0.09 mm

#### Data collection

Bruker SMART CCD area-detector diffractometer
Absorption correction: multi-scan (*SADABS*; Bruker, 2001 ▶) *T*_min_ = 0.652, *T*_max_ = 0.809
14917 measured reflections
3561 independent reflections
3468 reflections with *I* > 2σ(*I*)
*R*_int_ = 0.020

#### Refinement

*R*[*F* ^2^ > 2σ(*F* ^2^)] = 0.015
*wR*(*F* ^2^) = 0.043
*S* = 1.04
3561 reflections
305 parameters
H atoms treated by a mixture of independent and constrained refinement
Δρ_max_ = 0.33 e Å^−3^
Δρ_min_ = −0.45 e Å^−3^

### 

Data collection: *SMART* (Bruker, 2001 ▶); cell refinement: *SAINT* (Bruker, 2001 ▶); data reduction: *SAINT*; program(s) used to solve structure: *SHELXS97* (Sheldrick, 2008 ▶); program(s) used to refine structure: *SHELXL97* (Sheldrick, 2008 ▶); molecular graphics: *SHELXTL* (Sheldrick, 2008 ▶); software used to prepare material for publication: *SHELXTL*.

## Supplementary Material

Crystal structure: contains datablocks I, global. DOI: 10.1107/S1600536811012360/jh2277sup1.cif

Structure factors: contains datablocks I. DOI: 10.1107/S1600536811012360/jh2277Isup2.hkl

Additional supplementary materials:  crystallographic information; 3D view; checkCIF report

## Figures and Tables

**Table 1:** Selected geometric parameters (Å, °)

Pr1—O5	2.3835 (17)
Pr1—O4^i^	2.4193 (16)
Pr1—O7^ii^	2.4366 (16)
Pr1—O1	2.4407 (15)
Pr1—O10	2.4643 (18)
Pr1—O9	2.459 (2)
Pr1—N1	2.6484 (18)
Pr1—N2	2.677 (2)

Symmetry codes: (i) 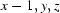; (ii) 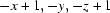.

**Table 2:** Hydrogen-bond geometry (Å, °)

*D*—H⋯*A*	*D*—H	H⋯*A*	*D*⋯*A*	*D*—H⋯*A*
N4—H4⋯O2^iv^	0.86	1.85	2.679 (3)	160
N3—H3*A*⋯O6^v^	0.86	1.88	2.735 (3)	172
N3—H3*A*⋯O5^v^	0.86	2.59	3.063 (3)	115
O9—H9*A*⋯O1^vi^	0.77 (3)	1.98 (3)	2.743 (3)	172 (3)
O10—H10*A*⋯O3^i^	0.88 (4)	1.77 (4)	2.645 (3)	173 (3)
O10—H10*A*⋯O4^i^	0.88 (4)	2.44 (3)	2.894 (3)	113 (3)
O10—H10*B*⋯O3^ii^	0.73 (4)	2.10 (4)	2.789 (3)	157 (4)
O9—H9*B*⋯O8^ii^	0.73 (3)	1.96 (4)	2.644 (3)	157 (3)

Symmetry codes: (i) 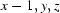; (ii) 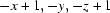; (iv) 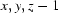; (v) 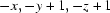; (vi) 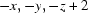.

**Fig. 1.**
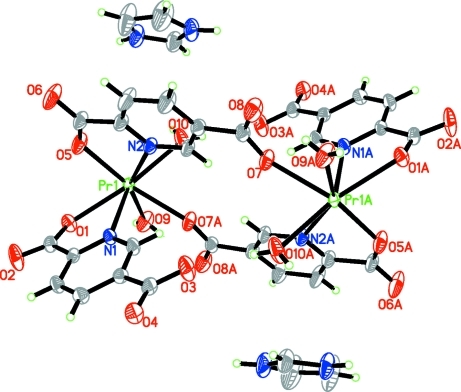
Molecular structure of 1 showing 50% probability displacement ellipsoids.

**Fig. 2.**
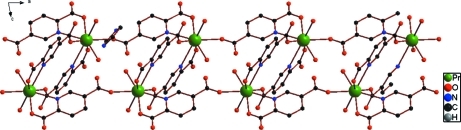
One-dimensional chain structure of 1 viewed down the b-axis

**Fig. 3.**
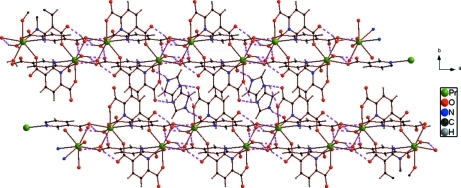
Hydrogen bonding packing diagram of 1 viewed down the b-axis.

## supplementary crystallographic information

### Comment

Pyridine-dicarboxylic acids as multidentate ligands containing N– and O– donors, have been widely used in preparing many kinds of coordination complexes especially in complexes containing rare earth metals (Chen *et al.* 2011, Zhao *et al.* 2009). Many complexes based on pyridine-2,5-dicarboxylic acid have been prepared (Li, Zhang *et al.* 2009, Li, Chen *et al.* 2009, Huang *et al.* 2009, Zhang *et al.* 2007, Zhang *et al.* 2005), but no complex containing Pr elememt, except two 3 d-4f complexes (Song *et al.* 2006, Chi *et al.* 2009) was reported. The reaction of pyridine-2,5-dicarboxylic acid with praseodymium salt under hydrothermal conditions results in the formation of a complex formulated as {(C_3_N_2_H_5_)[(C_7_H_3_NO_4_)_2_Pr(H_2_O)_2_]}_n_, and the complex has been structurally characterized by elemental analysis and X-ray diffraction. The structure of 1 viewed down the *b*-axis was presented in Fig. 2. Hydrogen banding packing diagram of 1 view down the *b*-axis was shown in Fig. 3. Selected band lengthes and angles were summarized inTable 1. The distances of the hydrogen bands were listed in Table 2.

### Experimental

The compound 1 was synthesized by solvothermal reaction. A mixture of pyridine-2,5-dicarboxylic acid (0.0334 g, 0.2 mmol), Pr(NO_3_)_3_.6H_2_O (0.0230 g, 0.05 mmol), imidazole (0.0361 g, 0.53 mmol) and water (3 ml) was sealed in a 7 ml glass tube and heated to 120 *^o^*C for 96 h. After cooling to room temperature, light green crystals were obtained.

### Refinement

Hydroxy H atoms were placed in calculated positions with O—H = 0.85 Å, and torsion angles were refined, Water H atoms were placed through fourier electronic density, *U*_iso_(H) = 1.5 *U*_eq_(O). Other H atoms were placed in calculated positions with C—H = 0.93 (aromatic and imidazole), N—H = 0.86 (imidazole)and refined in riding mode, with *U*_iso_(H) = 1.2 *U*_eq_(C or N).

### Crystal data

**Table:**

(C_3_H_5_N_2_)_2_[Pr_2_(C_7_H_3_NO_4_)_4_(H_2_O)_4_]	*Z* = 1
*M**_r_* = 1152.48	*F*(000) = 568
Triclinic, *P*1	char
Hall symbol: -p 1	*D*_x_ = 1.887 Mg m^−^^3^
*a* = 9.5444 (19) Å	Mo *K*α radiation, λ = 0.71073 Å
*b* = 10.667 (2) Å	Cell parameters from 3561 reflections
*c* = 11.222 (2) Å	θ = 2.6–25.5°
α = 64.63 (3)°	µ = 2.47 mm^−^^1^
β = 79.50 (3)°	*T* = 293 K
γ = 87.50 (3)°	Block, light green
*V* = 1014.3 (3) Å^3^	0.19 × 0.16 × 0.09 mm

### Data collection

**Table:**

Bruker SMART CCD area-detector diffractometer	3561 independent reflections
Radiation source: fine-focus sealed tube	3468 reflections with *I* > 2σ(*I*)
graphite	*R*_int_ = 0.020
ω scans	θ_max_ = 25.0°, θ_min_ = 2.0°
Absorption correction: multi-scan (*SADABS*; Bruker, 2001)	*h* = −11→11
*T*_min_ = 0.652, *T*_max_ = 0.809	*k* = −12→12
14917 measured reflections	*l* = −13→13

### Refinement

**Table:**

Refinement on *F*^2^	Primary atom site location: structure-invariant direct methods
Least-squares matrix: full	Secondary atom site location: difference Fourier map
*R*[*F*^2^ > 2σ(*F*^2^)] = 0.015	Hydrogen site location: inferred from neighbouring sites
*wR*(*F*^2^) = 0.043	H atoms treated by a mixture of independent and constrained refinement
*S* = 1.04	*w* = 1/[σ^2^(*F*_o_^2^) + (0.0271*P*)^2^ + 0.5143*P*] where *P* = (*F*_o_^2^ + 2*F*_c_^2^)/3
3561 reflections	(Δ/σ)_max_ = 0.068
305 parameters	Δρ_max_ = 0.33 e Å^−^^3^
0 restraints	Δρ_min_ = −0.45 e Å^−^^3^

### Special details

**Table:**

Geometry. All e.s.d.'s (except the e.s.d. in the dihedral angle between two l.s. planes) are estimated using the full covariance matrix. The cell e.s.d.'s are taken into account individually in the estimation of e.s.d.'s in distances, angles and torsion angles; correlations between e.s.d.'s in cell parameters are only used when they are defined by crystal symmetry. An approximate (isotropic) treatment of cell e.s.d.'s is used for estimating e.s.d.'s involving l.s. planes.
Refinement. Refinement of *F*^2^ against ALL reflections. The weighted *R*-factor *wR* and goodness of fit *S* are based on *F*^2^, conventional *R*-factors *R* are based on *F*, with *F* set to zero for negative *F*^2^. The threshold expression of *F*^2^ > σ(*F*^2^) is used only for calculating *R*-factors(gt) *etc*. and is not relevant to the choice of reflections for refinement. *R*-factors based on *F*^2^ are statistically about twice as large as those based on *F*, and *R*- factors based on ALL data will be even larger.

### Fractional atomic coordinates and isotropic or equivalent isotropic displacement parameters (Å^2^)

**Table:**

	*x*	*y*	*z*	*U*_iso_*/*U*_eq_	
Pr1	0.194718 (10)	0.107410 (10)	0.725374 (9)	0.01889 (5)	
O1	0.17790 (15)	0.14537 (16)	0.92701 (14)	0.0277 (3)	
N1	0.43859 (18)	0.13489 (18)	0.79437 (16)	0.0228 (4)	
O4	0.94311 (15)	0.15185 (18)	0.73503 (16)	0.0337 (4)	
N2	0.36969 (19)	0.25158 (18)	0.49260 (17)	0.0250 (4)	
C5	0.5686 (2)	0.1198 (2)	0.7350 (2)	0.0252 (4)	
H5	0.5761	0.0949	0.6642	0.030*	
O9	0.0720 (2)	−0.1109 (2)	0.89507 (19)	0.0386 (4)	
O5	0.19850 (19)	0.35430 (16)	0.63801 (16)	0.0379 (4)	
O10	0.1175 (2)	0.0591 (2)	0.5508 (2)	0.0473 (5)	
C7	0.8356 (2)	0.1232 (2)	0.7005 (2)	0.0263 (5)	
O3	0.83952 (17)	0.0841 (2)	0.60974 (18)	0.0429 (4)	
C1	0.4287 (2)	0.1698 (2)	0.89743 (19)	0.0205 (4)	
C6	0.2795 (2)	0.1837 (2)	0.9634 (2)	0.0248 (4)	
O6	0.2580 (2)	0.57179 (18)	0.49073 (19)	0.0560 (6)	
C2	0.5464 (2)	0.1908 (2)	0.9421 (2)	0.0269 (5)	
H2	0.5361	0.2152	1.0133	0.032*	
C4	0.6928 (2)	0.1394 (2)	0.7731 (2)	0.0223 (4)	
C8	0.3728 (2)	0.3897 (2)	0.4479 (2)	0.0291 (5)	
O2	0.26769 (19)	0.2280 (2)	1.0488 (2)	0.0498 (5)	
C13	0.2689 (3)	0.4449 (2)	0.5310 (2)	0.0325 (5)	
C3	0.6802 (2)	0.1750 (2)	0.8796 (2)	0.0264 (4)	
H3	0.7611	0.1882	0.9088	0.032*	
C12	0.4576 (2)	0.1982 (2)	0.4206 (2)	0.0263 (4)	
H12	0.4542	0.1025	0.4494	0.032*	
N4	0.0958 (3)	0.3420 (3)	0.1892 (2)	0.0493 (6)	
H4	0.1334	0.2930	0.1485	0.059*	
C15	−0.0147 (3)	0.3044 (3)	0.2871 (3)	0.0434 (6)	
H15	−0.0651	0.2196	0.3246	0.052*	
C17	0.0534 (4)	0.5118 (3)	0.2475 (4)	0.0757 (12)	
H17	0.0578	0.5961	0.2529	0.091*	
N3	−0.0426 (3)	0.4050 (2)	0.3233 (2)	0.0471 (6)	
H3A	−0.1110	0.4039	0.3854	0.056*	
C16	0.1408 (4)	0.4721 (3)	0.1631 (4)	0.0760 (12)	
H16	0.2177	0.5237	0.0987	0.091*	
C11	0.5533 (2)	0.2772 (2)	0.3059 (2)	0.0262 (5)	
C14	0.6545 (2)	0.2095 (2)	0.2339 (2)	0.0259 (4)	
C10	0.5552 (3)	0.4201 (3)	0.2616 (3)	0.0439 (7)	
H10	0.6176	0.4769	0.1845	0.053*	
C9	0.4634 (3)	0.4765 (2)	0.3334 (3)	0.0460 (7)	
H9	0.4624	0.5722	0.3051	0.055*	
O8	0.7309 (2)	0.28565 (18)	0.12655 (17)	0.0446 (5)	
O7	0.65553 (16)	0.07807 (15)	0.28965 (15)	0.0308 (3)	
H9A	0.002 (4)	−0.129 (3)	0.946 (3)	0.041 (8)*	
H10A	0.025 (4)	0.069 (3)	0.564 (3)	0.062 (10)*	
H10B	0.149 (4)	0.034 (4)	0.501 (4)	0.066 (12)*	
H9B	0.111 (4)	−0.174 (3)	0.904 (3)	0.047 (10)*	

### Atomic displacement parameters (Å^2^)

**Table:**

	*U*^11^	*U*^22^	*U*^33^	*U*^12^	*U*^13^	*U*^23^
Pr1	0.01230 (7)	0.02507 (7)	0.02203 (7)	0.00136 (5)	0.00114 (5)	−0.01435 (5)
O1	0.0164 (7)	0.0434 (9)	0.0278 (7)	−0.0005 (6)	0.0028 (6)	−0.0218 (7)
N1	0.0155 (8)	0.0325 (9)	0.0258 (8)	0.0028 (7)	−0.0017 (7)	−0.0184 (7)
O4	0.0152 (8)	0.0515 (10)	0.0452 (9)	0.0058 (7)	−0.0054 (7)	−0.0313 (8)
N2	0.0237 (9)	0.0243 (9)	0.0255 (8)	0.0045 (7)	0.0023 (7)	−0.0122 (7)
C5	0.0183 (11)	0.0369 (12)	0.0272 (10)	0.0040 (9)	−0.0011 (8)	−0.0213 (9)
O9	0.0263 (9)	0.0326 (10)	0.0447 (10)	0.0001 (8)	0.0163 (8)	−0.0139 (8)
O5	0.0420 (10)	0.0259 (8)	0.0365 (9)	0.0040 (7)	0.0156 (7)	−0.0139 (7)
O10	0.0226 (10)	0.0907 (16)	0.0575 (12)	0.0071 (9)	−0.0061 (8)	−0.0598 (12)
C7	0.0170 (11)	0.0327 (11)	0.0320 (11)	0.0055 (9)	−0.0022 (9)	−0.0178 (9)
O3	0.0201 (8)	0.0775 (13)	0.0533 (10)	0.0061 (8)	−0.0017 (7)	−0.0512 (10)
C1	0.0184 (10)	0.0224 (9)	0.0210 (9)	0.0024 (8)	0.0003 (8)	−0.0113 (8)
C6	0.0210 (11)	0.0292 (11)	0.0259 (10)	0.0025 (9)	0.0015 (8)	−0.0157 (9)
O6	0.0734 (14)	0.0263 (9)	0.0484 (11)	0.0104 (9)	0.0250 (10)	−0.0122 (8)
C2	0.0255 (11)	0.0359 (12)	0.0248 (10)	0.0018 (9)	−0.0016 (9)	−0.0192 (9)
C4	0.0166 (10)	0.0255 (10)	0.0240 (10)	0.0028 (8)	−0.0013 (8)	−0.0111 (8)
C8	0.0301 (12)	0.0260 (11)	0.0286 (11)	0.0062 (9)	0.0020 (9)	−0.0126 (9)
O2	0.0279 (9)	0.0867 (14)	0.0640 (12)	0.0029 (9)	0.0032 (8)	−0.0643 (12)
C13	0.0342 (13)	0.0275 (12)	0.0332 (11)	0.0086 (10)	0.0041 (10)	−0.0155 (10)
C3	0.0187 (10)	0.0351 (11)	0.0300 (10)	0.0024 (9)	−0.0059 (8)	−0.0177 (9)
C12	0.0277 (11)	0.0218 (10)	0.0274 (10)	0.0045 (9)	0.0025 (9)	−0.0117 (9)
N4	0.0505 (14)	0.0556 (14)	0.0455 (13)	0.0149 (12)	0.0073 (11)	−0.0326 (11)
C15	0.0455 (16)	0.0406 (14)	0.0449 (14)	0.0077 (12)	0.0026 (12)	−0.0239 (12)
C17	0.080 (3)	0.0472 (18)	0.089 (3)	−0.0075 (17)	0.039 (2)	−0.0394 (18)
N3	0.0485 (14)	0.0444 (12)	0.0450 (12)	0.0069 (11)	0.0155 (11)	−0.0259 (10)
C16	0.074 (2)	0.0543 (19)	0.078 (2)	−0.0050 (17)	0.0407 (19)	−0.0288 (17)
C11	0.0247 (11)	0.0277 (11)	0.0249 (10)	0.0054 (9)	0.0004 (9)	−0.0122 (9)
C14	0.0209 (11)	0.0324 (11)	0.0271 (10)	0.0040 (9)	0.0000 (9)	−0.0172 (9)
C10	0.0512 (16)	0.0282 (12)	0.0367 (13)	0.0020 (11)	0.0191 (12)	−0.0099 (10)
C9	0.0570 (18)	0.0217 (11)	0.0423 (14)	0.0075 (11)	0.0175 (13)	−0.0089 (10)
O8	0.0481 (11)	0.0357 (9)	0.0361 (9)	0.0047 (8)	0.0184 (8)	−0.0128 (7)
O7	0.0261 (8)	0.0275 (8)	0.0378 (8)	0.0042 (6)	0.0054 (7)	−0.0176 (7)

### Geometric parameters (Å, °)

**Table:**

Pr1—O5	2.3835 (17)	O6—C13	1.236 (3)
Pr1—O4^i^	2.4193 (16)	C2—C3	1.380 (3)
Pr1—O7^ii^	2.4366 (16)	C2—H2	0.9300
Pr1—O1	2.4407 (15)	C4—C3	1.385 (3)
Pr1—O10	2.4643 (18)	C8—C9	1.379 (3)
Pr1—O9	2.459 (2)	C8—C13	1.511 (3)
Pr1—N1	2.6484 (18)	C3—H3	0.9300
Pr1—N2	2.677 (2)	C12—C11	1.382 (3)
O1—C6	1.263 (3)	C12—H12	0.9300
N1—C5	1.335 (3)	N4—C15	1.310 (4)
N1—C1	1.346 (3)	N4—C16	1.361 (4)
O4—C7	1.253 (3)	N4—H4	0.8600
O4—Pr1^iii^	2.4193 (16)	C15—N3	1.303 (3)
N2—C8	1.337 (3)	C15—H15	0.9300
N2—C12	1.336 (3)	C17—C16	1.341 (4)
C5—C4	1.384 (3)	C17—N3	1.355 (4)
C5—H5	0.9300	C17—H17	0.9300
O9—H9A	0.77 (3)	N3—H3A	0.8600
O9—H9B	0.73 (3)	C16—H16	0.9300
O5—C13	1.263 (3)	C11—C10	1.386 (3)
O10—H10A	0.88 (4)	C11—C14	1.504 (3)
O10—H10B	0.73 (4)	C14—O8	1.240 (3)
C7—O3	1.249 (3)	C14—O7	1.267 (3)
C7—C4	1.499 (3)	C10—C9	1.376 (3)
C1—C2	1.373 (3)	C10—H10	0.9300
C1—C6	1.513 (3)	C9—H9	0.9300
C6—O2	1.224 (3)	O7—Pr1^ii^	2.4366 (16)
			
O5—Pr1—O4^i^	78.67 (7)	C2—C1—C6	121.25 (18)
O5—Pr1—O7^ii^	139.99 (6)	O2—C6—O1	125.8 (2)
O4^i^—Pr1—O7^ii^	135.10 (6)	O2—C6—C1	117.6 (2)
O5—Pr1—O1	77.58 (6)	O1—C6—C1	116.62 (17)
O4^i^—Pr1—O1	87.46 (6)	C1—C2—C3	119.03 (19)
O7^ii^—Pr1—O1	117.28 (6)	C1—C2—H2	120.5
O5—Pr1—O10	104.04 (8)	C3—C2—H2	120.5
O4^i^—Pr1—O10	72.69 (6)	C3—C4—C5	117.72 (19)
O7^ii^—Pr1—O10	75.16 (7)	C3—C4—C7	121.57 (19)
O1—Pr1—O10	159.11 (6)	C5—C4—C7	120.71 (18)
O5—Pr1—O9	145.52 (6)	N2—C8—C9	122.7 (2)
O4^i^—Pr1—O9	74.82 (7)	N2—C8—C13	115.37 (19)
O7^ii^—Pr1—O9	73.98 (6)	C9—C8—C13	121.9 (2)
O1—Pr1—O9	79.59 (6)	O6—C13—O5	125.2 (2)
O10—Pr1—O9	88.76 (8)	O6—C13—C8	119.1 (2)
O5—Pr1—N1	83.59 (7)	O5—C13—C8	115.67 (19)
O4^i^—Pr1—N1	148.64 (5)	C2—C3—C4	119.4 (2)
O7^ii^—Pr1—N1	73.00 (6)	C2—C3—H3	120.3
O1—Pr1—N1	63.42 (5)	C4—C3—H3	120.3
O10—Pr1—N1	137.32 (6)	N2—C12—C11	123.74 (19)
O9—Pr1—N1	108.60 (7)	N2—C12—H12	118.1
O5—Pr1—N2	62.40 (6)	C11—C12—H12	118.1
O4^i^—Pr1—N2	117.33 (6)	C15—N4—C16	108.2 (2)
O7^ii^—Pr1—N2	80.22 (6)	C15—N4—H4	125.9
O1—Pr1—N2	124.85 (5)	C16—N4—H4	125.9
O10—Pr1—N2	71.92 (7)	N3—C15—N4	109.0 (3)
O9—Pr1—N2	151.07 (6)	N3—C15—H15	125.5
N1—Pr1—N2	75.27 (6)	N4—C15—H15	125.5
C6—O1—Pr1	125.79 (12)	C16—C17—N3	106.5 (3)
C5—N1—C1	117.88 (18)	C16—C17—H17	126.8
C5—N1—Pr1	126.03 (13)	N3—C17—H17	126.8
C1—N1—Pr1	116.08 (13)	C15—N3—C17	109.1 (2)
C7—O4—Pr1^iii^	140.65 (14)	C15—N3—H3A	125.4
C8—N2—C12	117.46 (18)	C17—N3—H3A	125.4
C8—N2—Pr1	116.34 (13)	C17—C16—N4	107.2 (3)
C12—N2—Pr1	126.14 (13)	C17—C16—H16	126.4
N1—C5—C4	123.45 (19)	N4—C16—H16	126.4
N1—C5—H5	118.3	C12—C11—C10	117.9 (2)
C4—C5—H5	118.3	C12—C11—C14	120.9 (2)
Pr1—O9—H9A	134 (2)	C10—C11—C14	121.2 (2)
Pr1—O9—H9B	115 (3)	O8—C14—O7	125.3 (2)
H9A—O9—H9B	111 (3)	O8—C14—C11	118.0 (2)
C13—O5—Pr1	130.05 (14)	O7—C14—C11	116.74 (18)
Pr1—O10—H10A	103 (2)	C9—C10—C11	119.0 (2)
Pr1—O10—H10B	137 (3)	C9—C10—H10	120.5
H10A—O10—H10B	119 (4)	C11—C10—H10	120.5
O3—C7—O4	124.8 (2)	C10—C9—C8	119.3 (2)
O3—C7—C4	118.38 (19)	C10—C9—H9	120.4
O4—C7—C4	116.84 (19)	C8—C9—H9	120.4
N1—C1—C2	122.48 (18)	C14—O7—Pr1^ii^	138.92 (13)
N1—C1—C6	116.26 (18)		

Symmetry codes: (i) *x*−1, *y*, *z*; (ii) −*x*+1, −*y*, −*z*+1; (iii) *x*+1, *y*, *z*.

### Hydrogen-bond geometry (Å, °)

**Table:**

*D*—H···*A*	*D*—H	H···*A*	*D*···*A*	*D*—H···*A*
N4—H4···O2^iv^	0.86	1.85	2.679 (3)	160
N3—H3A···O6^v^	0.86	1.88	2.735 (3)	172
N3—H3A···O5^v^	0.86	2.59	3.063 (3)	115
O9—H9A···O1^vi^	0.77 (3)	1.98 (3)	2.743 (3)	172 (3)
O10—H10A···O3^i^	0.88 (4)	1.77 (4)	2.645 (3)	173 (3)
O10—H10A···O4^i^	0.88 (4)	2.44 (3)	2.894 (3)	113 (3)
O10—H10B···O3^ii^	0.73 (4)	2.10 (4)	2.789 (3)	157 (4)
O9—H9B···O8^ii^	0.73 (3)	1.96 (4)	2.644 (3)	157 (3)

Symmetry codes: (iv) *x*, *y*, *z*−1; (v) −*x*, −*y*+1, −*z*+1; (vi) −*x*, −*y*, −*z*+2; (i) *x*−1, *y*, *z*; (ii) −*x*+1, −*y*, −*z*+1.
